# Research Question, Objectives, and Endpoints in Clinical and Oncological Research: A Comprehensive Review

**DOI:** 10.7759/cureus.29575

**Published:** 2022-09-25

**Authors:** Addanki Purna singh, Praveen R Shahapur, Sabitha Vadakedath, Vallab Ganesh Bharadwaj, Dr Pranay Kumar, Venkata BharatKumar Pinnelli, Vikram Godishala, Venkataramana Kandi

**Affiliations:** 1 Physiology, Saint James School of Medicine, The Quarter, AIA; 2 Microbiology, Bijapur Lingayat District Educational Association (BLDE, Deemed to be University) Shri B.M. Patil Medical College, Vijayapur, IND; 3 Biochemistry, Prathima Institute of Medical Sciences, Karimnagar, IND; 4 Microbiology, Trichy Sri Ramasamy Memorial (SRM) Medical College Hospital & Research Centre, Tiruchirapalli, IND; 5 Anatomy, Prathima Institute of Medical Sciences, Karimnagar , IND; 6 Biochemistry, Vydehi Institute of Medical Science & Research Center, Bangalore, IND; 7 Biotechnology, Ganapathy Degree College, Parkal, IND; 8 Clinical Microbiology, Prathima Institute of Medical Sciences, Karimnagar, IND

**Keywords:** endpoints, objectives, research question, cancer, clinical research, covid-19, severe acute respiratory syndrome coronavirus-2

## Abstract

Clinical research is a systematic process of conducting research work to find solutions for human health-related problems. It is applied to understand the disease process and assist in the diagnosis, treatment, and prevention. Currently, we are experiencing global unrest caused by the coronavirus disease (COVID-19) pandemic. The novel severe acute respiratory syndrome coronavirus (SARS-CoV-2) has been responsible for the deaths of more than 50 million people worldwide. Also, it has resulted in severe morbidity among the affected population. The cause of such a huge amount of influence on human health by the pandemic was the unavailability of drugs and therapeutic interventions to treat and manage the disease. Cancer is a disease condition wherein the normal cell function is deranged, and the cells multiply in an uncontrolled manner. Based on recent reports by the World Health Organization (WHO), cancer is the second leading cause of death globally. Moreover, the rates of cancers have shown an increasing trend in the past decade. Therefore, it is essential to improve the understanding concerning clinical research to address the health concerns of humans. In this review, we comprehensively discuss critical aspects of clinical research that include the research question, research objectives, patient-reported outcome measures (PROMs), intention-to-treat and per-protocol analysis, and endpoints in clinical and oncological research.

## Introduction and background

Successful clinical research can be conducted by well-trained researchers. Other essential factors of clinical research include framing a research question and relevant objectives, documenting, and recording research outcomes, and outcome measures, sample size, and research methodology including the type of randomization, among others [1,2].

Clinicians/physicians and surgeons are increasingly dependent on the clinical research results for improved management of patients. Therefore, researchers need to work upon a relevant research question/hypothesis and specific objectives that may potentially deliver results that can be translated into practice in the form of evidence-based medicine [3].

Essential elements that facilitate a researcher to frame a research question are in-depth knowledge of the subject and identifying possible gaps. Moreover, the *feasible, interesting, novel, ethical, relevant* (FINER) approach and the *population of interest/target group, intervention, comparison group, outcome of interest, and time of study* (PICOT) approach were previously suggested for researchers to be able to frame appropriate research questions [4].

Moreover, the research objectives should be framed by the researcher before the initiation of the study: a *specific, measurable, achievable, realistic, and time-defined* (SMART) approach is utilized to devise the objectives based on the research question. It is preferable to have a single primary objective whereas the secondary objectives can be multiple and may be dependent on the amount of data collected. The objectives must be simple and specific and must reflect the research question [5,6].

Given the evolution and the increasing requirement for emergency care, clinical researchers are advised to adopt a* population, exposure, comparator, outcome* (PECO)/*population, exposure, comparator, outcome* (PICO) approach to construct the study objectives and carry out quantitative research. In contrast, qualitative research which is carried out to understand, explore, and examine requires the researcher to understand what and why the research is undertaken along with the roles of the researcher, research process/steps, and participants [7,8].

**Figure 1:**
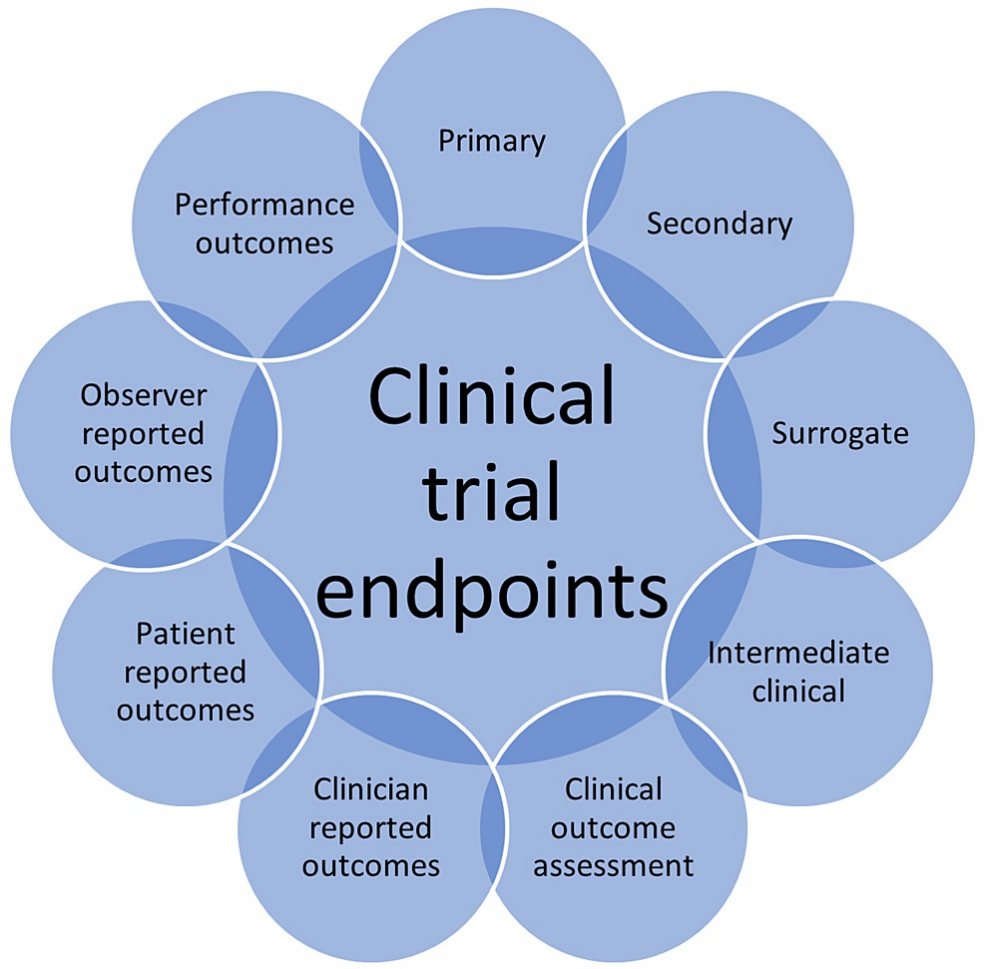
Clinical trial endpoints and outcomes This figure has been created by the authors

Since clinical research is envisaged in finding a solution to a health problem, choosing the appropriate endpoint requires special focus. The endpoints are the specific measures of the outcomes of an intervention and therefore they must be chosen judiciously [9]. The endpoints in a clinical trial can be single or multiple in numbers. The primary endpoints assess the major research question, and the secondary endpoints may assess alternative research questions. Moreover, there are other endpoints like surrogate endpoints, intermediate clinical endpoints, clinical outcomes, clinical outcome assessments, clinician-reported outcomes, observer-reported outcomes, patient-reported outcomes, and performance outcomes [10] (Figure 1 ).

The clinical trial endpoints are essentially the indicators of the power of the interventions either to cure or control the disease progression. Due to the cost and the tedious nature of the clinical trials, it is suggested that multiple-arm trials that include more than one primary endpoint be used [11]. Integration of the primary endpoints with the patient prioritized endpoints was recently suggested especially among cardiovascular disease patients [12]. Cancer research is an increasingly evolving area because of the unavailability of therapeutic interventions for some malignancies like breast and lung cancer, among others [13,14]. Moreover, the drugs available for treating cancers are plagued by adverse reactions. However, since most cancer clinical trials apply overall survival as the preferred and gold standard clinical endpoint, it is difficult for the trial operators to sustain the costs associated with the long lengths of the study that follow-up patients for years to assess the clinical outcomes after interventions. In this study, we comprehensively review the essential elements of clinical research that include the research question, hypothesis, and clinical and oncological endpoints.

## Review

Research question

A research question can alternatively be called the aim of the researcher. It describes the problem that the researcher intends to solve vis-à-vis finding an answer to a question. A research question is the first step toward any kind of research process that includes both qualitative as well as quantitative research. Since the research question predicts the core of any project, it must be carefully framed. The essential elements to consider while determining a research question are feasibility, preciseness, and relevance to the real world.

A person interested in a broad subject area must first complete extensive reading of the available literature. This enables the researcher to find out the strengths, loopholes, deficiencies, and missing links that can form the basis for framing a research question. The problem to which a solution needs to be found and the potential causes/reasons for the problems help a researcher frame the research question.

**Figure 2:**
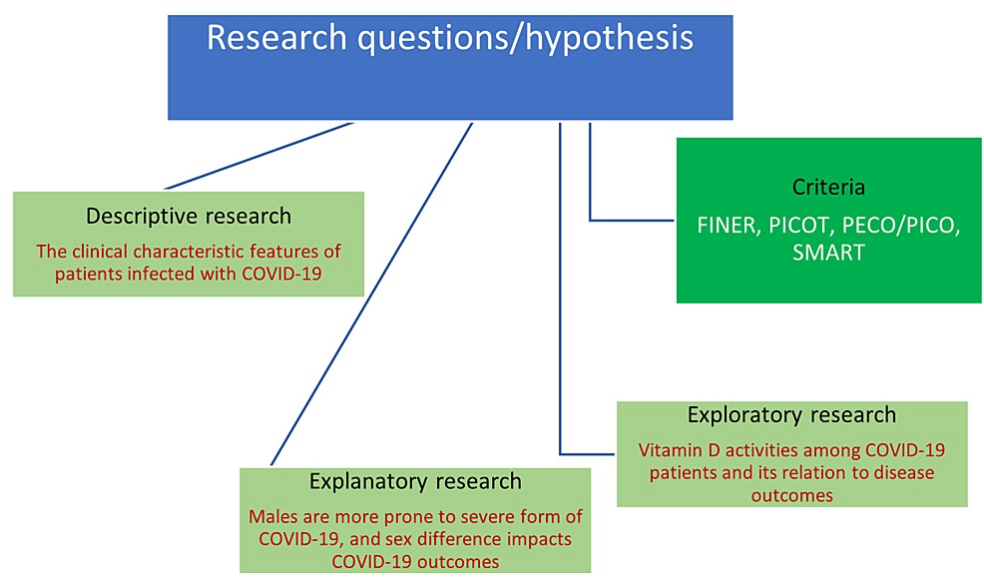
Diagrammatic representation of framing research questions based on research type FINER: Feasible, interesting, novel, ethical, relevant; PICOT: Population of interest/target group, intervention, comparison group, outcome of interest, time of study; PECO: Population, exposure, comparator, outcome; PICO: Population, intervention, comparator, outcome; SMART: Specific, measurable, achievable, realistic, and time defined; COVID-19: Coronavirus disease-19 This figure has been created by the authors

The research questions should be framed in such a way that the researcher will find several possibilities as solutions to the research question rather than a simple yes or no. Among the various factors that determine the power of a research question, the most essential ones include the ability of research questions to find complex answers, focussed, and the specific nature of the question. The research questions must be answerable, debatable, and researchable [15,16]. Research questions differ from the type of research method selected by the researcher as shown in Figure 2.

Hypothesis testing

A hypothesis is an assumption by the researcher that the answers drawn with reference to the research question are either true or false. The researcher performs hypothesis testing by using appropriate statistical methods on the data collected from the research.

The hypothesis is an assumption/observation of the researcher regarding the outcome of potential research that is being conducted. There are two types of hypotheses, the null hypothesis (H0), wherein, the researcher assumes that there is no relation/causality/effect. The alternate hypothesis (HA) is when the researcher believes/assumes that there is a relationship/effect [17]. Basically, there are two types of errors while testing a hypothesis. Type I error (α) (false positive) is when the researcher rejects the null hypothesis although it is true. Type II error (β) (false negative) is when the researcher accepts the null hypothesis although it is false.

The errors in hypothesis testing occur because of bias among many other reasons in the study. Many studies set the power of the studies to essentially rule out errors. Researchers consider 5% chance (α=0.05; range: 0.01-0.10) of error in case of a type I error and up to 20% chance (β =0.20; range: 0.05-0.20) in case of type II errors [18]. The characteristics of a good hypothesis are simple and specific. Moreover, it must be decided by the researcher prior to initiating the study and while writing the study proposal/protocol [18]. 

Hypothesis testing means sample testing, wherein the information gathered after sample testing is inferred after applying statistical methods. A hypothesis may be generated in several ways that include observations, anatomical features, and other physiological facts observed by physicians [19]. Hypothesis testing also can be performed by using appropriate statistical methods. The testing of the hypothesis is done to prove the null hypothesis or otherwise use the sample data.

As a researcher, one must assume a null hypothesis, or believe that the alternate hypothesis holds good in the sample selected. After the collection of data, analysis, and interpretation, the researcher either accepts or rejects the hypothesis. Therefore, it must be noted that while a study is initiated, there is only a 50% chance of either the null hypothesis or the alternative hypothesis coming true [20].

The step-by-step process of hypothesis testing starts with an assumption, criteria for interpretation of results, analysis, and conclusion. The level of significance (95% free of type I error and 80% free of type II error) also is decided initially to ensure that the study results are replicated by the other researchers [21].

Objectives in clinical research

The most significant objective in clinical research is to find out whether the intervention attempted was successful in curing the disease/medical condition. It is important to understand the fact that research planning greatly influences the research results, and no statistical method can improve the results but a well-designed and conducted clinical research [22].

The primary objective of clinical research studies includes improvement in patient management. Most clinical research studies are aimed at discovering a new/novel drug to treat a medical condition that presently has no specific treatment, or the available drugs are not particularly effective in curing the disease.

**Figure 3:**
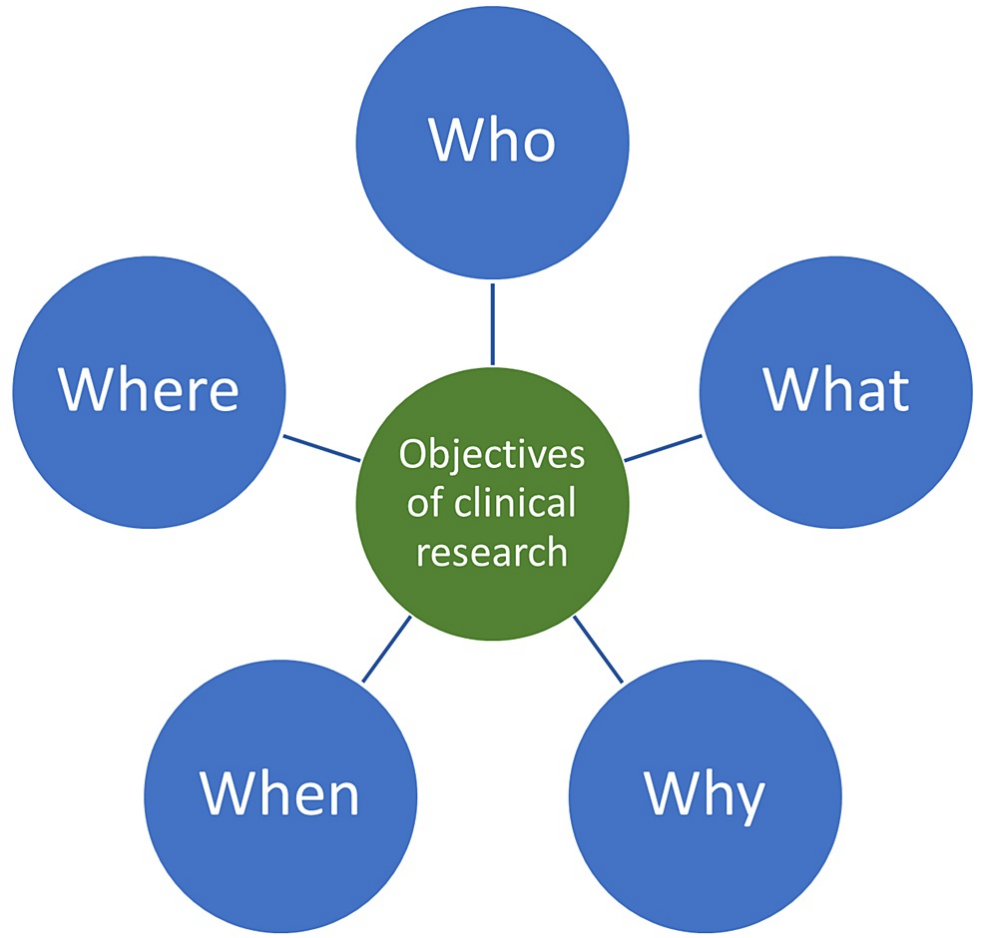
The objectives of clinical research This figure has been created by the authors

The objectives are formed to address the five 'W's, namely who (children, women, etc.); what (the medical condition/disease/infection); why (causes of the medical condition/disease/infection); when (conditions responsible for the medical condition/disease/infection); and where (geographical aspects of the medical condition/disease/infection) as shown in Figure 3.

Clinical research can be of several types including primary research and secondary research. Also, the research can be observational (no intervention) and experimental/interventional. Clearly demarcated/framed research objectives are essential to improve the clarity, specificity, and focus of the clinical trial [23].

Patient-reported outcome measures (PROMs)

While conducting clinical research the investigators collect trial data through clinical observations, laboratory monitoring, and caregiver feedback. There are some aspects of the data like the patient-reported outcomes (PROs) that can be reported by the subject/patient him/herself either in the form of a questionnaire or through interviews. Such data collected from the patients in their words is termed PROMs. The PROMs include a global impression of the trial, the functional status and well-being, symptoms, health-related quality of life (HRQL), treatment compliance, and satisfaction. 

The questionnaire used to collect the PROs is called a PRO instrument. The data collected through this instrument is used to establish the benefit-to-risk ratio of the clinical trial drug. The PRO instruments can be designed as generic (contains a wide variety of health-related aspects and therefore can be used among different patient types), disease-specific (rheumatoid arthritis, psoriasis, etc.), dimension-specific (physical activity, cognitive levels, etc.), region/site-specific, individualized, utility measures, and summary items [24]. The patient-reported experience measure (PREMs), and the patient and public involvement programs are used to collect the patient’s feedback that invariably helps in improving the quality of healthcare facilities [25].

It is important to develop PROM tools/instruments for various diseases, especially among children as noted by a recent research report. This study suggested that a suitable PROM instrument is required to measure the status of disease (wheezing/asthma/respiratory diseases) and its control among preschool children [26].

Intention to treat and per-protocol analysis

The intention to treat (ITT) analysis is used when all the study subject’s data is analyzed including all those participants who were enrolled in the study, and those who have deviated (not signed the informed consent, discontinued from the study, not taken the trial drug as suggested). The ITT studies minimize the bias and ensure both the study and the control groups are compared.

The per-protocol (PP) analysis usually includes the data from only those subjects who have remained till the study period ended, have taken the drugs as suggested by the protocol, and was available for regular follow-up. The disadvantages of PP are potential disturbances in the balance between the study groups (randomized/placebo/control), a lower number of study participants due to the exclusion of dropouts, and non-compliant subjects. Therefore, the results from the PPA studies could be biased [27].

The randomized clinical trial (RCT) studies of superiority type use ITT analyses as against the non-inferiority and equivalence studies wherein an ITT approach may favor the study hypothesis.

According to the Committee for Proprietary Medicinal Products (CPMP), and the Consolidated Standards for reporting trials (CONSORT), both the ITT and PP analyses must be assessed to effectively interpret the results of the clinical trial including the safety and efficacy [28]. 

Endpoints in clinical research

The endpoints in clinical research determine whether the clinical trial has been successful in finding out if an intervention/drug has proven beneficial in improving the survival and quality of life of the patient. The endpoints determine the validity of the clinical trial results. There are different types of endpoints like primary endpoints, secondary endpoints, tertiary endpoints, surrogate endpoints (laboratory measurements, physical signs), and others [9].

The clinical trial endpoints could be subjective and objective in nature. The objective endpoints are survival, disease progression/remission, and the development of disease/condition. The subjective endpoints include symptoms, quality of life, and other patient-reported outcomes [29,30]. The significance of endpoints in clinical trials and the importance of choosing appropriate endpoints were previously reported. This study suggested that the primary endpoints help in deriving the sample size and confirm the generalizability of the results. The secondary and surrogate endpoints could be used while conducting the clinical research without ignoring the aspects of the quality of life of the subjects [31]. 

Endpoints in oncology clinical trials

Cancer clinical trials assume increased significance because the drugs developed against the cancer are intended to increase the survival of the patients. Also, anti-cancer agents are associated with several side effects. Therefore, oncology trials include several endpoints, the primary being the overall survival, and the secondary endpoints include the assessment of other outcomes that indicate the quality of life (QoL), tumor-related endpoints, and others. The disadvantages of oncology clinical trials are the cost associated with the recruitment of a greater number of subjects and long-time follow-up of the patients [32].

Although primary endpoints are considered as most significant in oncological trials, a recent report stressed the importance of surrogate markers in assessing the efficacy of anti-cancer drugs [33]. 

The endpoints in oncology clinical trials, their applications, functions, and drawbacks are summarized in Table 1.

**Table 1:** The endpoints in oncology clinical trials, their applications, functions, and drawbacks

Endpoint	Applications	Functions	Drawbacks
Overall survival	Gold standard primary oncological endpoint	Assesses time from randomization to death, clinical benefit, easily measurable, gives definite results, and eliminates researcher bias	Not effective in slowly progressing diseases, requires quite a high patient number, influenced by cross-over, subsequent therapies, and non-cancer deaths
Duration of clinical benefit	Primary endpoint	Assesses time from randomization to disease progression or death in patients who achieve a complete response, partial response, or stable disease for 24 weeks or longer	Needs disease-specific validation
Complete response	A primary endpoint that can also be used as a surrogate endpoint	Assesses time from randomization to survival advantage associated with improved overall survival and prolonged event-free survival in specific treatment studies	Needs disease-specific validation
Time to treatment failure	A primary endpoint when used in conjunction with secondary endpoints	Assesses the time from the initiation of chemotherapy treatment/intervention to its early discontinuation	The inclusion of older patients may affect the results
Health-related quality of life	Preferred as a secondary clinical endpoint	Assesses patient’s quality of life with respect to health status over time, can directly measure the patient’s benefit	Data may frequently be missing, and inadequate, the clinical relevance of exceedingly small changes is unknown and requires multiple analyses and validation
Progression-free survival	A surrogate marker for regular and accelerated approval	Assesses time from randomization until the first evidence of disease progression or death, requires a limited patient number, short follow-up period, objective and quantitative evaluation, cost-effective, and not influenced by crossovers or subsequent therapies	It cannot be statistically validated as a surrogate marker for survival, is not definitely measurable, is subject-dependent with a high risk of bias, definitions may differ between studies, and the time of evaluation needs to be balanced between treatment arms
Disease-free survival	A surrogate marker for regular and accelerated approval	Assesses time from randomization until evidence of disease recurrence, and requires limited patient number and short follow-up	It cannot be statistically validated as a surrogate marker for survival, not definitely measurable, and definitions may differ between studies
Objective response rate	A surrogate marker for regular and accelerated approval	Assesses how a specific treatment impacts tumor burden in a patient with a history of solid tumors, needs to be evaluated in single-arm studies, much quicker evaluation as compared with survival studies, and requires much more limited patient number	Benefits cannot be measured directly, and detailed measurement of drug activity is unavailable
Duration of response	Used as a surrogate marker	Assesses time from randomization to disease progression or death in patients who achieve complete or partial response	Needs disease-specific validation
Pathological complete response	Preferred as a surrogate marker	Assesses time from randomization to absence of residual invasive cancer upon evaluation of the resected breast tissue and regional lymph nodes	Disease-specific, especially in breast cancer
Disease control rate	Clinical benefit for survival	Assesses time from randomization to complete response, partial response, or stable disease	Can exaggerate the anticancer effect of the therapy
Clinical benefit rate	Clinical benefit for survival	Assesses time from randomization to complete response, partial response, or at least six months of stable disease	It does not necessarily measure clinical benefit
Milestone survival	Used as a surrogate endpoint/qualitative endpoint	Assesses time from randomization to survival probability at a given time point	Requires further validation
Time to progression	Preferred as a surrogate and not a primary endpoint	Assesses time from randomization to first evidence of disease progression and effectiveness of targeted therapy	Can be adversely affected by patients’ disease characteristics
Event free survival	Preferred as a surrogate and as an alternative to the primary endpoint	Assesses time from randomization to disease progression, discontinuation of the treatment, and/or death	It needs to be validated for each unique disease/tumor type, treatment, and stage of disease
Time to next treatment	Preferred as a surrogate endpoint for incurable diseases	Follows up the treatment response until the initiation of next-line therapy	Requires validations for specific disease

Survival endpoints in oncology clinical trials

The survival endpoint considers the time from randomization to death. This type of follow-up (daily), although difficult to do, will remove bias associated with the investigator’s interpretation. The survival studies require large sample sizes and cross-over therapies act as confounding factors for survival. The survival studies consider patient benefit over drug toxicity. 

Apart from the overall survival, oncology clinical trials use alternative ways to assess the efficacy of the drugs by using other endpoints like progression-free survival [34]. Other endpoints suggested are biomarkers, disease-free survival, objective response rate, time to progression, complete response, partial response, minor response, time to treatment failure, time to next treatment, duration of clinical benefit, objective response rate, complete response, pathological complete response, disease control rate, clinical benefit rate, milestone survival, event-free survival, and QoL [35].

Endpoints in immunological diseases and infections

Autoimmune diseases are usually chronic conditions that arise due to the immunologic responses against the self. They are usually associated with hyper-reactivity of immune cells towards the host's own cells/tissue and can cause significant morbidity and mortality among affected people.

Autoimmune diseases are generally genetic in origin, but many such diseases are attributed to other factors such as infection, food, drugs, and other substances. Frequently occurring autoimmune diseases are rheumatoid arthritis, psoriasis, systemic lupus erythematosus (SLE), ulcerative colitis, Crohn’s disease, and multiple sclerosis, among others [36].

Clinical trials with respect to the development of drugs/medicine to treat autoimmune diseases take into consideration the therapeutic efficacy of the trial drug, no risk, and only benefit to the patients. Therefore, the selection of endpoints for clinical trials in immune diseases must consider all these factors to effectively assess the pharmacological value of the trial drugs. The endpoints for autoimmune hepatitis include remission, incomplete response, treatment failure, and drug toxicity [37].

The endpoint related to the infections includes the direct measurement of the number of microorganisms. Other endpoints include the measurement of physiological aspects impaired by the infecting microbe and the measurement of immune responses against the infectious agent. The endpoints of human immunodeficiency virus (HIV) infection includes HIV-ribonucleic acid (RNA) viral load, maintenance, improvement, and decline of the cluster of differentiation 4 (CD4)+ T lymphocyte cell counts, and others.

Composite endpoints

Clinical trial for drugs is assessed based on several endpoints that establish efficacy and allow regulatory authorities to decide on approving the drugs for human use. In most instances, the clinical trials apply primary endpoints whereas in recent times the surge in candidate drugs and the necessity for life-saving drugs had ushered in the use of alternative endpoints like the composite endpoints. The composite endpoints are instrumental in reducing the trial costs, minimizing the long follow-ups, and lower subject recruitments. Since the composite endpoints combine more than one outcome during the drug trial, it enables the investigators to understand the efficacy of the drug in a short period of time [38].

While using multiple endpoints, it is important to understand that each one is as important as the other and the statistical methods must be used to confirm the overall efficacy of the trial drug. The drawbacks of applying composite endpoints in clinical trials are the complexity of the methods, low transparency, including invalid indicators, and the possibility of misleading results and conclusions [39].

Because there is no specific recommendation as to how the composite endpoints need to be selected, evaluated, and analyzed, there exists a possibility of bias. In a recent report, an index to evaluate the bias attributable to composite outcomes (BACO) was applied and suggested. The BACO index <0, 0 to <1, and >1 indicated that the composite endpoints were inverted, underestimated, and overestimated, respectively. A BACO index of 1 indicates that the composite endpoint usage resulted in unbiased results [40]. The composite endpoint in a clinical trial means the use of multiple endpoints. For the drug trials for migraine, the composite endpoints include no pain for two hours, nausea after two hours, and photosensitivity after two hours. The clinical trials with vaccines could have more than 20 endpoints. Other trials with multiple endpoints include rheumatoid arthritis (4), acne (4), sleep disorders (6), primary biliary cirrhosis (4), and glaucoma (9).

## Conclusions

The clinical research design must be carefully accomplished keeping in mind the financial and time constraints. The trial must be initiated to address a specific research question, which essentially requires the research group to carry out an extensive literature search and identify the knowledge gaps. The research hypothesis needs to be carefully designed to avoid errors. The researchers should ensure the inclusion of appropriate objectives that guarantee quality outcomes and clinical benefits. It is essential to include the PROMs along with the clinician, researcher, and observer reported outcomes to assess the benefit-to-risk ratio of the investigational drug and improve the quality of healthcare facilities, among others. The safety and efficacy of a clinical trial drug should be carefully interpreted based on the results obtained from the ITT and PP analyses. Moreover, a clinical trial should incorporate specific and relevant endpoints that ensure the efficacy or otherwise of the intervention. Cancer clinical trials are even more complex because the interventions could potentially be life-saving and therefore, the selection of endpoints becomes critical as discussed briefly in this review.
